# Single-cell Technologies in Atherosclerosis: Uncovering Cellular Heterogeneity, Mechanisms, and Therapeutic Opportunities

**DOI:** 10.1007/s11883-026-01432-0

**Published:** 2026-06-18

**Authors:** Carlos V. Serrano, Bruna S. Matuck, Joao A. C. Lima

**Affiliations:** 1 Einstein Hospital Israelita, São Paulo, SP Brazil; 2 Department of Cardiopneumology, Instituto Do Coracao HCFMUSP (InCor), Faculty of Medicine, University of Sao Paulo, Av. Eneas C. de Aguiar 44 – Building 2, Floor 2, Room 2, Sao Paulo, SP 05403-904 Brazil; 3 Department of Medicine, Division of Cardiology, Johns Hopkins University, Baltimore, MD USA; 4 Department of Medicine, Division of Cardiovascular Medicine, Brigham and Women’s Hospital, Harvard Medical School, Boston, MA USA

**Keywords:** Atherosclerosis, Single-cell RNA sequencing (scRNA-seq), Spatial transcriptomics, Cellular heterogeneity, Precision medicine

## Abstract

**Purpose of Review:**

This review highlights recent advances in single-cell analysis technologies and their application in clarifying the cellular and molecular complexity of atherosclerosis, redefining our understanding of vascular biology and immune cell functioning within the atherosclerotic plaque.

**Recent Findings:**

Single-cell RNA sequencing (scRNA-seq), single-cell ATAC-seq, and spatial transcriptomics have revealed an unforeseen diversity of immune and vascular cell states in human and experimental prototypes of atherosclerosis. These techniques have led to the finding of novel macrophage and smooth muscle cell (SMC) phenotypes, distinct endothelial dysfunction signatures, and oligoclonal T cell populations. By integrating transcriptomic, epigenomic, proteomic, and spatial data, researchers have clarified key mechanisms of disease progression and identified cell-specific molecular pathways responsive to targeted therapy.

**Summary:**

Single-cell methods are changing our understanding of atherosclerosis by determining the heterogeneity, plasticity, and functional states of plaque-resident cells. These understandings contribute for the development of novel biomarkers, precision diagnostics, and targeted immunomodulatory strategies, with the ultimate goal of improving risk stratification and personalized treatment in atherosclerotic cardiovascular disease.

## Introduction: a New Era in Atherosclerosis Research

Atherosclerosis, characterized by lipid accumulation and chronic inflammation in medium and large arteries, remains the leading cause of cardiovascular morbidity and mortality worldwide, primarily through its contribution to myocardial infarction, stroke, and peripheral arterial disease. Traditionally regarded as a lipid-storage disorder, atherosclerosis is now well established as a chronic, immune-mediated inflammatory disease, driven by complex interactions among vascular, immune, and stromal cells [1, 2]. Recent advances, particularly in molecular and cellular biology, have significantly deepened our understanding of the mechanisms underlying atherosclerotic pathogenesis [3].

The limited access to substantial human atherosclerotic tissue constrains a comprehensive understanding of the functional and biological state of individual cell populations. This limitation reduces our ability to fully characterize cellular heterogeneity, rare cell types, and dynamic phenotypic transitions happening through plaque progression and regression [4]. The advent of single-cell omics technologies – particularly scRNA-seq, single-cell ATAC-seq (scATAC-seq), and spatially resolved transcriptomics – has transfigured cardiovascular research by allowing unbiased profiling of individual cells within diseased tissues (Table 1). These technologies provide high-resolution maps of the cellular architecture of atherosclerotic plaques, yielding unprecedented insight into pathogenic mechanisms and therapeutic opportunities [5].

**Table 1:** Key single-cell and spatial transcriptomic technologies used in atherosclerosis research. These technologies enable detailed characterization of cellular heterogeneity, regulatory mechanisms, and spatial organization within atherosclerotic plaques [39]

Technology	Primary output	Applications in atherosclerosis
scRNA-seq	Gene expression at single-cell level	Identify cell subtypes and states
scATAC-seq	Chromatin accessibility per cell	Map regulatory regions and TF networks
CITE-seq	mRNA and protein expression	Link transcriptomics with surface phenotype
Spatial Transcriptomics	Spatial gene expression in tissue sections	Preserve tissue context and map interactions

*scRNA-seq* Single-cell RNA sequencing – measures gene expression at the single-cell level; *scATAC-seq*; Single-cell Assay for Transposase-Accessible Chromatin using sequencing – identifies chromatin accessibility per cell; *CITE-seq* Cellular Indexing of Transcriptomes and Epitopes by sequencing – simultaneously captures mRNA and surface protein expression; *TF* Transcription factor – proteins that regulate gene expression; Spatial Transcriptomics: Technique to quantify gene expression while preserving spatial tissue architecture

## Advances in Single-cell and Spatial Technologies

### Single-cell Transcriptomics and Epigenomics

Among emerging technologies, scRNA-seq allows the high-resolution profiling of thousands of individual cells, revealing their gene-expression programs in an unbiased and systematic manner. In atherosclerosis, scRNA-seq has uncovered previously unknown subtypes of macrophages, SMCs, endothelial cells (ECs), and T cells, each with potentially distinct roles in inflammation, lipid metabolism, and matrix remodeling. These findings challenge conventional categorizations and point to a previously unsuspected degree of cellular heterogeneity and dynamic cellular interactions [6].

Additionally, scATAC-seq generates important regulatory maps. This technique provides knowledge on chromatin accessibility, offering insight into regulatory elements, enhancer-promoter interactions, and the activity of transcription factors. When applied to atherosclerotic tissues, scATAC-seq has confirmed key epigenetic regulators of lineage reprogramming, such as KLF4 and TCF21, which control SMC plasticity and macrophage activation [7]. Such gene regulatory networks are critical patterns for understanding gene regulation and the intricate interactions that drive biological processes. Recent advances have highlighted the potential of integrating scATAC-seq to scRNA-seq in offering unprecedented insights into how chromatin accessibility plays an important part in gene regulation and its role in pathological transitions [8].

### Multiplexed Protein and Multimodal Profiling

Multiplexed imaging plataforms such as Imaging Mass Cytometry (IMC), CODEX, and Phenocycler allow simultaneous detection of dozens of protein markers. These tools enhance immune phenotyping and validate transcriptional data. Emerging single-cell multi-omics technologies have evolved to integrate multiple molecular modalities simultaneously, encompassing the transcriptome, genome, epigenome, epitranscriptome, proteome, and metabolome. These techniques bridge the gap between transcriptomic and proteomic data enhancing cell state resolution and facilitate better functional annotation of immune and vascular cell populations [9, 10].

Recent progresses in technologies, like REAP-seq and ECCITE-seq, allow simultaneous measurement of proteins and mRNAs in single cells. Hopefully, molecular data on clonotype information (e.g., T-cell receptor (TCR) / B-cell receptor (BCR) sequences, perturbations (e.g., CRISPR-based screens), or protein secretion are obtained [11]. These capabilities expand the scope of single-cell analysis from observational to mechanistic studies, potentially accelerating drug discovery and biomarker elaboration. The diversity of TCRs and BCRs underpins the adaptive immune system’s ability to recognize and respond to a wide array of antigens. Recent advancements in RNA sequencing have expanded its application beyond transcriptomics to include the analysis of immune repertoires, enabling the exploration of TCR and BCR sequences across various physiological and pathological contexts, such as atherosclerosis [12].

### Spatial Omics

A critical limitation of dissociative single-cell techniques is the loss of spatial context, which obscures the spatial relationships between cells and their microenvironment. Spatial transcriptomics overcomes this drawback by preserving tissue architecture while allowing high-resolution gene expression outlining. This methodology provides a more integrated view of cellular heterogeneity, intercellular interactions, and tissue architecture [13].

Spatial transcriptomics technologies can be broadly categorized into sequencing based and probe-based approaches. While sequencing based platforms such as Visium (10 × Genomics), Stereo seq (BGI), and Aviti (Element Biosciences) enable transcriptome wide profiling with high sensitivity, they often lack single cell or subcellular resolution, limiting their ability to precisely map cellular interactions within complex tissues.

In contrast, probe-based platforms such as Xenium (10 × Genomics), CosMx SMI (NanoString), and MERSCOPE (Vizgen) offer subcellular resolution and enable highly multiplexed detection of targeted transcripts, making them particularly well suited for dissecting the spatial organization of cell states and interactions within the tissue microenvironment.

When applied to atherosclerotic plaques and perivascular regions, these high-resolution imaging tools allow detailed characterization of cellular heterogeneity – such as macrophage-rich necrotic cores or endothelial clusters undergoing endothelial-to-mesenchymal transition (EndMT) – and how they are spatially distributed and engage in paracrine signaling, identification of rare or novel cell states, and spatial mapping of receptor–ligand interactions. Such analyses can uncover key mechanistic pathways involved in disease progression and immune modulation in atherosclerosis [14, 15].

Spatial proteomics is a multidimensional technique that studies the spatial distribution and function of proteins within cells or tissues across both spatial and temporal dimensions. This approach complements transcriptomic data and allows for the direct visualization of protein expression within intact tissue sections, providing insights into the cellular and molecular organization of atherosclerotic plaques. Compared to transcriptomics, protein markers are more stable and better defined for immune cell phenotyping, which is particularly important in the inflamed and heterogeneous environment of atherosclerosis. This consideration makes spatial proteomics a more robust approach for identifying and classifying immune and stromal populations involved in plaque progression and remodeling.

In addition to functional profiling, spatial proteomics plays a critical role in cell segmentation. The use of membrane and nuclear markers enables accurate definition of single-cell boundaries, which is essential for generating high-quality single-cell spatial datasets. Proper segmentation ensures that molecular signals are assigned to the correct cell, which is crucial for downstream analyses such as cell–cell interaction mapping and neighborhood analysis.

While spatial transcriptomics provides broader coverage of gene expression, spatial proteomics offers higher spatial resolution and improved accuracy in defining cell types and states. In the context of atherosclerosis, where immune infiltration, endothelial dysfunction, and smooth muscle plasticity occur in highly organized microenvironments, spatial proteomics provides a more precise and functionally relevant view of the cellular landscape driving disease. Combining scRNAseq data and spatial transcriptomics enable the generation of high-resolution maps of human atherosclerotic plaques [1].

### Future Directions in Spatial Analyses

Single-slide spatial multiomics enables the simultaneous analysis of RNA and protein expression within the same tissue section, preserving spatial context while integrating molecular modalities. This approach enhances cell type annotation by leveraging the stability and specificity of protein markers for accurate segmentation, particularly in immune and vascular cells. Once cells are segmented and classified based on proteomic data, transcriptomic profiles can be used to reveal transcriptional heterogeneity and functional states within each population. In the context of atherosclerosis, this strategy is powerful, allowing the identification of distinct immune and stromal subsets and enabling the reconstruction of cell–cell communication networks. The ability to assign molecular identity and interaction potential within the same spatial framework makes single slide multiomics a pivotal tool for elucidating the cellular architecture and signaling landscapes of complex tissue microenvironments.

## Revisiting the Cellular Players in Atherogenesis

**Fig. 1:**
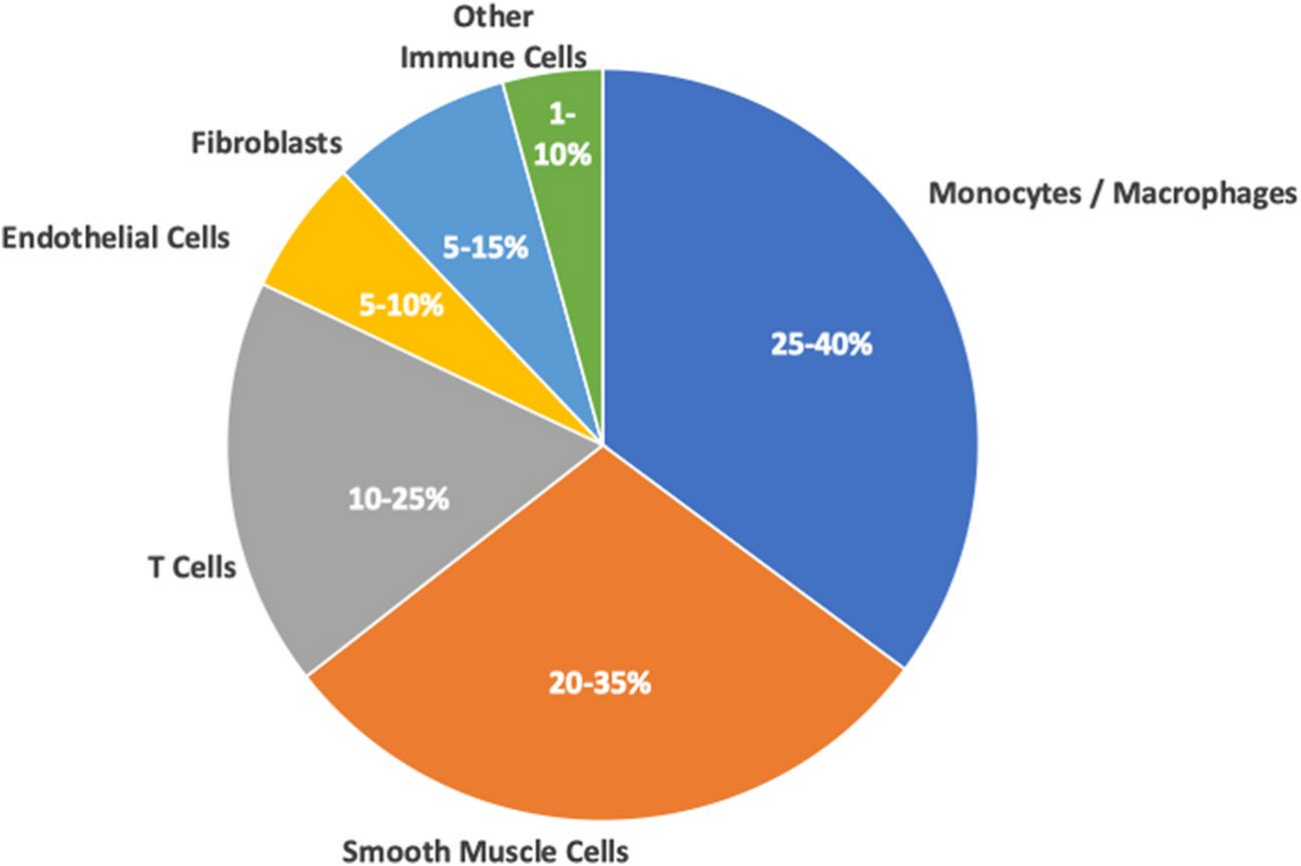
Cellular composition in atherosclerotic plaques. This pie chart illustrates the relative proportions of major cell types present in atherosclerotic plaques based on single-cell analysis. The dominant populations include macrophages, which play a central role in inflammation, lipid uptake, foam cell formation, efferocytosis, and smooth muscle cells, key contributors to plaque structure and stability and transdifferentiation. T cells represent the adaptive immune response within the lesion and pro- and anti-inflammatory cytokine release. Fibroblasts and endothelial cells are involved in extracellular matrix remodeling and vascular integrity as well as leukocyte recruitment, respectively. Other immune cells, such as B cells, dendritic cells, mast cells and NK cells, constitute a smaller but relevant component of the immune microenvironment within plaques [37, 38]

Relative proportions of major cell types within atherosclerotic plaques, as revealed by single-cell analysis, highlight macrophages and smooth muscle cells as predominant populations (Fig. 1).

### Macrophages: a Spectrum of Inflammatory States

Macrophages are highly adaptable immune cells capable of responding dynamically to diverse environmental signals. They are essential in maintaining local homeostasis, coordinating immune responses, enabling tissue repair, and, under certain conditions, contributing to disease pathogenesis such as in atherosclerosis [1]. Macrophages are central to atherogenesis, contributing to lipid uptake, inflammation, efferocytosis, and arterial remodeling. Single-cell profiling has dismantled the binary M1/M2 classification, revealing instead a spectrum of transcriptionally distinct macrophage subsets. These include pro-inflammatory macrophages expressing IL1β and NLRP3, lipid-laden foam cells marked by TREM2 and CD9, and reparative macrophages with high expression of anti-inflammatory genes like MRC1 and ARG1 [16].

In both human and murine atherosclerotic plaques, TREM2 + foam cell macrophages represent a conserved, disease-associated cellular population involved in lipid handling and phagocytosis. Other subsets specialize in antigen presentation (expressing MHC-II genes), iron handling (SPIC +), or extracellular matrix degradation (MMP9 +). Their relative abundance and functional activity appear to correlate with atherosclerotic disease stage and clinical outcomes [17].

### T Cells: Expanded Clonotypes and Functional Diversity

T lymphocytes infiltrate plaques and modulate inflammation by means of cytokine secretion and cell–cell interactions [18]. Single-cell analyses, coupled with TCR sequencing, have confirmed oligoclonal expansions of CD8 + and CD4 + T cells, suggesting chronic antigen-driven inflammatory responses within the arterial wall. These T cells express markers of exhaustion (PD-1, LAG3) and cytotoxicity (GZMB, PRF1), and some appear to be tissue-resident [19].

Regulatory T cells (Tregs), described by FOXP3 expression, play a crucial anti-inflammatory role but are often functionally compromised or numerically reduced in high-risk plaques. Th1 and Th17 subsets, producers of IFN-γ and IL-17 respectively, determine pro-inflammatory pathways and associate with plaque instability. Spatial analyses suggest that T cells localize preferentially near macrophages, suggesting direct immunomodulatory crosstalk [19].

### Endothelial Cells: from Quiescence to Mesenchymal Transition

Endothelial cells pave the vascular lumen and act as “gatekeepers” of vascular homeostasis. In atherosclerosis, ECs undergo structural and functional modifications in response to impaired flow, oxidative stress, and inflammation. Recent studies have provided a detailed single-cell atlas of ECs with description of EC subtypes with disparity expression of adhesion molecules (VCAM1, ICAM1), angiogenic mediators (ANGPT2, VEGFA), and markers of endothelial-to-mesenchymal transition (EndMT), such as SNAI1, ZEB2, and ACTA2 [20].

Importantly, EndMT is being acknowledged as a contributor to plaque fibrosis and calcification. It represents a pathological transdifferentiation of ECs into mesenchymal-like cells with reduced barrier function and increased matrix production. Targeting EndMT actions may offer a therapeutic approach to restore endothelial function [21].

### Smooth Muscle Cells: Phenotypic Modulation and Plasticity

SMCs exhibit considerable phenotypic plasticity in atherosclerosis. Lineage-tracing studies have demonstrated that medial SMCs can lose contractile markers (e.g., MYH11, ACTA2) and assume macrophage-like, osteogenic, or fibroblast-like identities. These transitions are supervised by transcriptional regulators such as KLF4, OCT4, and TCF21, and can have both protective and deleterious consequences depending on context [22, 23].

For instance, fibromyocyte-like SMCs contribute to the fibrous cap and plaque stability, while macrophage-like SMCs may exacerbate inflammation and lipid retention. Understanding the signals and regulators of SMC destiny outcomes may prove critical to formulating therapies that stimulate plaque stabilization [24].

### Adventitia and Perivascular Adipose Tissue

While usually unnoticed, the adventitia and perivascular adipose tissue (PVAT) are gradually valued as immunologically active compartments [25]. Single-cell profiling of these sections has identified resident dendritic cells, fibroblast subsets, and innate lymphoid cells that secrete cytokines and influence vascular inflammation [26].

Adventitial fibroblasts may attend as progenitors for myofibroblasts or SMC-like cells, contributing to vascular remodeling. PVAT, depending on its phenotype (brown vs. white-like), can promote anti- or pro-inflammatory effects through adipokine secretion, inducing systemic metabolism and local inflammation [27]. The adventicia also harbors connections with the nervous system, a subject of much recent interest [28].

## Cell–cell Communication and Regulatory Networks

The dynamic performance of cells within plaques is coordinated by a complex intercellular communication. Computational tools such as CellPhoneDB, NicheNet, and CellChat analyze single-cell data to deduce ligand-receptor interactions between cell types. These analyses have reaffirmed important cell communications – such as CCL2-CCR2, TNF-TNFRSF1A, and CD40-CD40LG – implicated in leukocyte recruitment, activation, and endurance [29].

Macrophage-T cell crosstalk modulates effector responses and immune frontier expression. ECs and SMCs interact through Notch and TGF-β signaling, prompting phenotypic transitions and fibrotic remodeling. Mapping these networks offers targets for therapeutic interruption of pathogenic loops or for improvement of protective circuits [30].

## Translational Implications and Clinical Relevance

### Biomarkers of Risk and Disease Activity

Single-cell signatures are being investigated as diagnostic and prognostic biomarkers. For example, detection of circulating monocytes with transcriptional profiles resembling inflammatory plaque macrophages may reflect active disease. Similarly, plasma-derived extracellular vesicles or cell-free RNA with single-cell-informed markers could serve as minimally invasive biomarkers [31]. Importantly, incorporating these molecular biomarkers with advanced non-invasive imaging modalities – such as ultra-high-resolution computed tomography – may significantly improve the early detection and monitoring of coronary atherosclerotic plaque formation and progression [32, 33].

### Targeted and Immunomodulatory Therapies

Cell-specific targets revealed by single-cell profiling offer a beginning for precision therapies. These include monoclonal antibodies or small molecules targeting pro-inflammatory macrophage pathways (e.g., IL-1β, NLRP3), SMC transition regulators (KLF4), or T cell checkpoints (PD-1, CTLA-4), or innate danger signals such as CD47, oxidized LDL, and reactive oxygen species [34]. Approaches that expand Treg populations or promote reparative macrophage phenotypes are under investigation [35].

### Integration with Imaging: Precision Risk Stratification

Merging high-resolution coronary imaging (e.g., optical coherence tomography, photon-counting coronary CT angiography) with single-cell insights permits better characterization of vulnerable plaques. Imaging of immune cell infiltration or molecular markers, informed by spatial omics findings, could improve precise risk stratification and therapeutic decision-making [31, 36].

### Challenges and Future Directions for Single-cell Analysis in Atherosclerosis

Despite transformative progress and expansion, several challenges persist [1]:

- **Standardization**: Proper organization of single-cell protocols, quality control procedures, and analytical workflows are crucial for ensuring reproducibility and minimizing batch effects that complicate data integration.
- **Clinical integration**: Linking the gap between experimental information and clinical efficacy requires validation in large, prospective cohorts.
- **Longitudinal data**: Time-course single-cell profiling during disease progression or treatment response are essential to identify atherosclerotic pathogenetic mechanisms.
- **Real-time imaging**: Advance of *in vivo* single-cell imaging transcriptomics would facilitate translation into interventional cardiology.
- **Cost and accessibility**: Prevalent clinical acceptance requires cost-effective platforms and simplified roadmaps suitable for routine diagnostics.

## Conclusion

Advances in single-cell and spatial omics have unshared a new era in cardiovascular research. By unraveling the intricate cellular landscape of atherosclerotic lesions, these tools provide unparalleled insights into disease mechanisms, cellular plasticity, and intercellular communications. The integration of multi-omic, spatial, and imaging data is laying the basis for a future of personalized, precision-based cardiovascular medicine. Continued technological innovation and collaborative translational research will be essential to completely realize their clinical potential.

## Key References

- Williams JW, Winkels H, Durant CP, Zaitsev K, Ghosheh Y, Ley K. Single Cell RNA Sequencing in Atherosclerosis Research. Circ Res. 2020;126(9):1112–1126. 10.1161/CIRCRESAHA.119.315940.
  - ○ The article by Williams et al. (2020) reviews the application of single-cell RNA sequencing (scRNA-seq) in atherosclerosis research. It highlights how scRNA-seq has revolutionized the understanding of cellular heterogeneity within atherosclerotic plaques, revealing distinct subsets of immune and non-immune cells. The authors discuss key discoveries, including novel macrophage and smooth muscle cell phenotypes with roles in inflammation, lipid handling, and fibrous cap formation. The review also emphasizes the importance of spatial context, integration with other omics, and the challenges of data interpretation. Ultimately, the authors underscore scRNA-seq as a transformative tool for uncovering mechanisms of disease and identifying new therapeutic targets in atherosclerosis.
  - Gastanadui MG, Margaroli C, Litovsky S, Richter RP, Wang D, Xing D, Wells JM, Gaggar A, Nanda V, Patel RP, Payne GA. Spatial Transcriptomic Approach to Understanding Coronary Atherosclerotic Plaque Stability. Arterioscler Thromb Vasc Biol. 2024;44(11):e264-e276. 10.1161/ATVBAHA.123.320330.
  - Gastanadui et al. (2024) applied spatial transcriptomics to human coronary atherosclerotic plaques to investigate molecular signatures associated with plaque stability and instability. The study revealed distinct spatial patterns of gene expression linked to inflammatory activity, extracellular matrix remodeling, and cellular composition within stable versus unstable regions. These findings provide understandings into the local microenvironment of plaques and highlight potential molecular targets for improving cardiovascular risk stratification and therapy.
- Adachi Y, Ueda K, Takimoto E. Perivascular adipose tissue in vascular pathologies-a novel therapeutic target for atherosclerotic disease? Front Cardiovasc Med. 2023;10:1151717. 10.3389/fcvm.2023.1151717.
  - ○ Adachi et al. (2023) review the role of perivascular adipose tissue (PVAT) – the fat surrounding most large blood vessels – in vascular health, describing its homeostatic vasculoprotective effects in healthy conditions and its shift to a pro-inflammatory source in settings like metabolic disease, aging, and chronic inflammation. They highlight molecular insights, including beiging of PVAT that exerts anti-inflammatory effects via factors such as NRG4, contrasted with later-stage PVAT fibrosis, reduced UCP1 expression, and increased cytokines like IL‑1β and IL‑6 that promote atherosclerosis. The authors propose that targeting PVAT – through omics-guided approaches or therapies such as GLP‑1 receptor agonists and SGLT2 inhibitors that modulate PVAT/EAT – could represent a novel therapeutic avenue to prevent or treat atherosclerotic disease.
- Schuijf JD, Lima JAC, Boedeker KL, Takagi H, Tanaka R, Yoshioka K, Arbab-Zadeh A. CT imaging with ultra-high-resolution: Opportunities for cardiovascular imaging in clinical practice. J Cardiovasc Comput Tomogr. 2022;16(5):388–396. 10.1016/j.jcct.2022.02.003.
  - ○ Schuijf et al. (2022) review how ultra‑high‑resolution CT (UHRCT) – with improved spatial resolution down to approximately 0.20-0.25 mm slice thickness – can overcome limitations of conventional CT, particularly in accurately imaging calcified plaques, stented segments, and small coronary vessels. The authors highlight early clinical evidence demonstrating that UHRCT reduces artifacts at calcified lesions and enables assessment of in‑stent lumens (especially in stents ≥ 2.5 mm) with greater diagnostic precision than conventional CT. They posit that wider adoption of UHRCT techniques may enhance non‑invasive characterization of coronary atherosclerosis and potentially inform clinical decision‑making and understanding of vascular pathophysiology.
- Pandit R, Yurdagul A. The Atherosclerotic Plaque Microenvironment as a Therapeutic Target. Curr Atheroscler Rep. 2025;27(1):47. https://doi.org/10.1007/s11883-025–01294-y.
  - ○ Pandit and Yurdagul test the traditional lipid‑centric model of atherosclerosis by highlighting how the plaque microenvironment – comprising extracellular matrix (ECM), soluble factors, and biomechanical forces – critically shapes lesion progression. They describe how transitional ECM proteins like fibronectin, especially in regions of disturbed flow, prime endothelial cells for inflammation and contribute to chronic plaque instability. Impaired efferocytosis – the clearance of dead cells by macrophages – leads to necrotic core expansion and worsens inflammatory signaling, further destabilizing lesions. The authors argue that therapeutic strategies targeting the local niche – such as enhancing macrophage efferocytosis, modulating integrin‑mediated endothelial activation, or delivering targeted agents via nanocarriers – could stabilize plaques and reduce vulnerability. Ultimately, they propose a shift toward interventions that engineer the plaque microenvironment, including ECM remodeling and precision delivery of pro‑resolving signals, as promising avenues to prevent acute cardiovascular events.

## Data Availability

No datasets were generated or analysed during the current study.

## Abbreviations

scRNA-seq: Single-cell RNA sequencing
SMC: Smooth muscle cell
scATAC-seq: Single-cell ATAC-seq
ECs: Endothelial cells
Tregs: Regulatory T cells
EndMT: Endothelial-to-mesenchymal transition
ILCs: Innate lymphoid cells
NIRF: Near-infrared fluorescence
PVAT: Perivascular adipose tissue
